# Vitamin D in Type 2 Diabetes and Its Correlation With Heat Shock Protein 70, Ferric Reducing Ability of Plasma, Advanced Oxidation Protein Products and Advanced Glycation End Products

**DOI:** 10.1002/edm2.508

**Published:** 2024-07-12

**Authors:** Nazanin Hashemi, Sahar Karimpour Reyhan, Reihane Qahremani, Kiana Seifouri, Meraj Tavakoli, Seyed Arsalan Seyedi, Farahnaz Ghaemi, Mahsa Abbaszadeh, Alireza Esteghamati, Manouchehr Nakhjavani, Hossein Mirmiranpour, Soghra Rabizadeh

**Affiliations:** ^1^ Endocrinology and Metabolism Research Center (EMRC), Vali‐Asr Hospital Tehran University of Medical Sciences Tehran Iran; ^2^ Department of Biology, Kerman Branch Islamic Azad University Kerman Iran

**Keywords:** AOPP, FRAP, HSP70, inflammation, Type 2 diabetes, vitamin D

## Abstract

**Aim:**

To investigate the association between vitamin D3 level and oxidative stress biomarkers such as Heat Shock Protein 70 (HSP70), ferric reducing ability of plasma (FRAP), advanced oxidation protein products (AOPP) and advanced glycation end products (AGEs) in patients with Type 2 diabetes.

**Method:**

In this cross‐sectional study, 54 patients including 32 females and 22 males with a mean age of 54.92 ± 11.37 years with T2D attending the diabetes clinic from 2021 to 2022 were included. According to the average level of vitamin D in this population (14.91), they were divided into two groups with vitamin D ≤15 ng/mL and vitamin D >15 ng/mL. Multivariate regression analysis was conducted to evaluate the relationship between vitamin D and AOPP, HSP and FRAP parameters. The correlation between vitamin D and other variables was evaluated via the Pearson correlation test.

**Result:**

Vitamin D level had a positive relation with FRAP (*β* = 0.32, *p* = 0.017) and HSP (*β* = 0.39, *p* = 0.003), but had a negative relation with AOPP (*β* = −0.30, *p* = 0.02). The level of 2hPP also had a negative relation with the level of vitamin D (*β* = −0.33, *p* = 0.03). There was not any relationship between the level of vitamin D and AGEs or other variables. After adjusting for multiple confounders for the multivariate regression model, HSP remained significant.

**Conclusion:**

This research indicates the relationship between vitamin D levels and oxidative stress biomarkers in patients with Type 2 diabetes.

## Graphical abstract

Vitamin D3 possesses antioxidant properties and reduces inflammation; as a result, it reduces insulin resistance and intracellular oxidative stress. In our study, vitamin D level had a positive correlation with FRAP and HSP, but had a negative correlation with AOPP. Therefore, vitamin D supplementation can be considered as an effective therapeutic intervention in diabetes control.

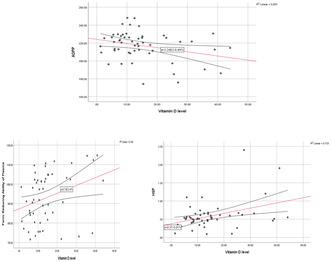

## 1 Introduction

Vitamin D deficiency is a global health problem and compared with the general population, patients with Type 2 diabetes (T2D) have a higher prevalence of vitamin D deficiency [1, 2, 3, 4]. Lower vitamin D levels have been associated with an increased risk of insulin resistance and metabolic syndrome and T2D in prospective observational studies [3, 5, 6]. Vitamin D3 possesses antioxidant properties and reduces inflammation; as a result, it reduces insulin resistance and intracellular oxidative stress (OS) [7, 8].

OS plays an important role in the pathogenesis of T2D and its complications. It is the result of an imbalance between the production of free radicals and the function of the antioxidative defence system, which can lead to an increase in insulin resistance and T2D through multiple molecular mechanisms, including pancreatic beta cell dysfunction [6, 7, 8].

Different biomarkers are used to study OS and its effects. Heat Shock Protein 70 (HSP70) is a molecular chaperone that can be intracellular (iHSP70) or extracellular (eHSP70). iHSP70 is essential in cell protection against stress‐induced damage and is anti‐inflammatory [9, 10]. Also, increased expression of iHSP70 against OS induced by glucotoxicity is one of the protective mechanisms of cells against the potential damage of OS. While in contrast, eHSP70 induces the activation of multiple proinflammatory pathways. As a result, chronic exposure to eHSP70 activates proinflammatory pathways likely by binding to membrane Toll‐like receptors. Although eHSP70‐peptides have also anti‐inflammatory and immunosuppressive action after internalization and antigen processing [10]. eHSP70 expression is induced in response to chronic inflammatory conditions and insulin resistance [11]. Potentially, the eHSP70/iHSP70 ratio can predict whether insulin resistance will eventually progress to diabetes or not [10]. In vitro studies showed that decreased HSP70 gene expression was associated with reduced expression of vitamin D receptor (VDR)‐dependent genes [12]. Vitamin D supplementation was accompanied by enhanced HSP70 expression and cell protection against OS [13]. Based on the available knowledge, one of the aims of this study was to evaluate the relationship between the levels of HSP70 and vitamin D in patients with T2D.

Higher levels of AOPPs (advanced oxidation protein products) have been identified in patients with diabetes, cardiovascular diseases, high blood pressure and atherosclerosis [14, 15]. AOPPs are structurally similar to advanced glycation end products (AGEs) and also exhibit biological activity, as evidenced by their ability to induce proinflammatory cytokines and adhesion molecules [16]. In an inflammatory state, higher levels of AOPP were reported in patients with hypovitaminosis D [17].

AGE production rate is up to 14‐fold higher in a hyperglycaemic state [18, 19]. AGEs affect all proteins, but long‐lived proteins with low turnover, which are already susceptible to cumulative damages over time, are more prone [18]. Faster unfavourable structural and functional alterations in cellular proteins put patients with diabetes at higher risk of developing diabetic complications. Different studies have shown a modifying effect of vitamin D on the level and impact of AGEs [20].

Ferric reducing ability of plasma (FRAP) is an index of the antioxidant capacity of plasma. Patients with T2D have decreased levels of FRAP. As the duration of T2D increases and diabetic complications appear, FRAP decreases more [21]. According to previous studies, vitamin D supplementation can be an effective intervention to decrease OS [22].

Type 2 DM is associated with high inflammatory burden [23], and vitamin D deficiency triggers chronic inflammation [24]. Indeed, diabetic subjects with a low vitamin D level are more likely to have poor controlled disease [25]. HSP70 involves in inflammatory conditions [26]; furthermore, AGEs are increased in diseases characterised with inflammation [27]. Hence, studying these markers in association with vitamin D and Type 2 DM is logical. Comprehension of the underlying pathophysiology will help us establish novel therapeutic interventions to improve the health and outcomes of patients with T2D in the future.

## 2 Method

### 2.1 Study Population

In this cross‐sectional study, 54 patients with T2D attending the diabetes clinic of Vali‐Asr Hospital affiliated with Tehran University of Medical Sciences from 2021 to 2022 were included. Diabetes was diagnosed based on the 2021 American Diabetes Association guidelines [28]. The inclusion criteria were diabetes, and age between 18 and 70. All patients were treated with oral agents. Exclusion criteria were a diabetic complication, cancer history, recent pregnancy, history of cirrhosis any chronic disorder except diabetes, recent use of vitamin D3 supplementation, recent blood transfusion, history of any acute or chronic inflammatory condition and end‐stage renal disease (ESRD). According to the average level of vitamin D in this population (14.91), they were divided into two groups with vitamin D ≤15 ng/mL and vitamin D >15 ng/mL.

### 2.2 Data Collection

The study was carried out based on the principles of the Declaration of Helsinki. Written informed consent was collected from all patients. The ethics committee of Tehran University of Medical Sciences confirmed the protocol of the study with the ethical code of IR.TUMS.IKHC.REC.1400.255.

Demographic and anthropometric data such as age, gender, height, weight, duration of diabetes and drug history were documented.

For measuring the height, patients stood ahead of the standard height board and the closest 0.1 cm was reported. Weight was measured using a digital scale and reported with 0.1‐kg accuracy. Body mass index (BMI) was calculated by dividing weight (in kilograms) by the square of height (in meters). After 15 min of sitting, blood pressure was measured via an automated blood pressure device. Two blood pressures were measured with a time interval of 10 min between them, and their average was recorded.

The subjects' serums were collected after 12 h of fasting and assessed with kits confirmed by the central reference laboratory. Laboratory data including fasting blood glucose (FBS), haemoglobin A1C (HbA1c), creatinine, urea, uric acid, glomerular filtration rate (GFR), triglyceride (TG), cholesterol (Chol), low‐density lipoprotein cholesterol (LDL‐c), high‐density lipoprotein cholesterol (HDL‐c), aspartate aminotransferase (AST), alanine aminotransferase (ALT), alkaline phosphatase (ALP), vitamin D3 level, advanced glycated end products (AGEs), protein products advanced oxidation (AOPP), fluorescence recovery after photobleaching (FRAP) and HSP70 were measured. HbA1c was analysed via high‐performance liquid chromatography (A1c, DS5 Pink Kit; Drew, Marseille, France). Creatinine was calculated by enzymatic method on an automated analyser. GFR was measured by the Modification of Diet in Renal Disease (MDRD) equation. FBS was measured by enzymatic colorimetric methods with the glucose oxidase test. Lipid profiles (TG, HDL‐C, LDL‐C) were measured by enzymatic procedures.

Serum HSP70 was determined via quantitative sandwich ELISA immunoassay (EKS‐715, stressgen, USA).

The AOPP of serum was measured with spectrophotometric methods (FLUOstar OPTIMA, BMG, Germany) as explained by Kalousova, Skrha, and Zima [29]. In this procedure, the concentration of 200 of serum is reduced by a factor of 5 in phosphate‐buffered saline (PBS). Also, 200 μL of chloramine‐T (0–100 mmol/L) was used for measurement and 200 μL of PBS was also added to different microplates as blank. For preparation, 10 μL of acetic acid and 20 μL of 1.16 M potassium iodide (KI) are added. In total, 82.3–232.7 μmol/L was considered as normal range. The intraassay and intraassay coefficient of variation were <5% and <10%, respectively.

FRAP was determined with spectrophotometry as expressed by Benzie and Strain [30]. According to this method, 300 mmol/L of acetate buffer (pH: 3.6), 10 mmol/L of tripyridyl triazine (TPTZ) in 40 mmol/L HCL and 20 mmol/L FeCl_3_·6H_2_O were mixed to prepare FRAP reagent. Twenty‐five microliter of serum and 750 μL FRAP reagent are mixed, and absorbance is determined at 593 nm. In total, 612–1634 μmol/L was considered as normal range. The intraassay and intraassay coefficient of variation were 3% and 4.2%, respectively.

AGEs were estimated by the spectrophotometric method of Kalousova, Skrha, and Zima [29]. The serum was diluted in PBS by a factor of 50. Fluorescence intensity at 350 nm excitation and 440 nm emission was measured and recorded as the percentage of fluorescent emission. The intraassay and intraassay coefficient of variation were 5.1% and 7.9%, respectively.

### 2.3 Statistical Analysis

All analyses were conducted via 24th version of SPSS software for Windows, and a *p* value <0.05 was considered statistically significant. We performed Shapiro–Wilk normality test to evaluate the normal distribution of the study population. Continuous variables with a normal distribution and without a normal distribution were recorded as Means ± standard deviations (SD) and interquartile range, respectively. *t*‐test was used to compare these variables between two groups of patients with vitamin D ≤15 ng/mL and vitamin D >15 ng/mL. Categorical variables were presented as frequencies or percentages, and for further analysis, chi‐square was performed. The correlation between vitamin D and other variables was evaluated via the Pearson correlation test. Multivariate regression analysis was conducted to evaluate the relationship between vitamin D and AOPP, HSP and FRAP parameters. Multiple confounders like age, sex, and metabolic indices such as blood pressure and lipid profile were adjusted in this analysis.

## 3 Results

In this study, 54 patients including 32 females and 22 males with a mean age of 54.92 ± 11.37 years were examined to investigate the relationship between the level of serum vitamin D and OS biomarkers (AGEs, FRAP, HSP, AOPP). The mean serum vitamin D level was 14.9 ± 10.42 ng/mL (1.20–44.10).

The patients were divided into two groups based on their serum vitamin D levels. Vitamin D level was lower than 15 ng/mL in 66.7% of patients, and in 33.3%, it was more than 15 ng/mL.

Table 1 shows the comparison of variables between the two groups. Mean BMI was 27.63 ± 4.30 in patients with vitamin D level more than 15 ng/dL and 27.85 ± 4.30 in patients with vitamin D level less than 15 ng/dL (*p* value = 0.84). In the group with vitamin D more than 15 ng/mL, patients had mean SBP of 129.22 ± 18.59 and in patients with vitamin D level less than 15 ng/dL SBP was 129.66 ± 21.01 which was not statistically significant (*p* value >0.05). In patients who had vitamin D level less than 15 ng/dL, mean FBS level and HbA1c level were 179.06 ± 58.20 and 7.61 ± 1.53, respectively, and the numbers in the other group were 166.35 ± 63.59 and 7.24 ± 2.24; which were not statistically different. Mean LDL‐c was 98.93 ± 43.43 in patients with vitamin D more than 15 ng/dL and 100.75 ± 30.68 in the other group, which was not significantly different either (*p* value >0.05). In patients with vitamin D more than 15 ng/mL, a lower level of AOPP (208 vs. 221, *p* = 0.003) was observed, but the level of FRAP (989 vs. 907, *p* = 0.04) and HSP (0.74 vs. 0.50, *p* = 0.02) was higher. There was no statistically significant difference in the level of AGEs between the two groups.

**TABLE 1:** Comparison of variables between two groups, based on serum vitamin D level.

Variables	Vitamin D >15 ng/mL 33.3% (*N* = 18)	Vitamin D <15 ng/mL 66.7% (*N* = 36)	*p*
Age (year)	56.11 ± 10.69	54.69 ± 12.97	0.69
Sex (female/male) (%)	64.5/35.5	63.6/36.4	0.94
BMI (kg/m^2^)	27.63 ± 4.44	27.85 ± 4.30	0.84
SBP (mmHg)	129.22 ± 18.59	129.66 ± 21.01	0.94
DBP (mmHg)	77.38 ± 12.57	78.13 ± 12	0.83
FBS (mg/dL)	166.35 ± 63.59	179.06 ± 58.20	0.48
HbA1C (%)	7.74 ± 2.24	7.61 ± 1.53	0.80
Uric acid (mg/L)	5.80 ± 1.27	5 ± 1.32	0.22
Urea	34.17 ± 14.94	28.15 ± 11.41	0.19
Creatinine (mg/dL)	0.92 ± 0.15	0.97 ± 0.24	0.52
Cholesterol (mg/dL)	179 ± 46.41	189.46 ± 47.32	0.48
LDL‐C (mg/dL)	98.93 ± 43.43	100.75 ± 30.68	0.87
HDL‐C (mg/dL)	41.06 ± 9.33	48.20 ± 23.72	0.27
Triglyceride (mg/dL)	150.66 ± 68.48	185.34 ± 91.47	0.20
AST (IU/L)	26.69 ± 11.27	19.38 ± 7.12	0.03
ALT (IU/L)	26.50 ± 14.19	20.27 ± 13.41	0.23
HSP70 (ng/mL)	0.74 ± 0.56	0.50 ± 0.21	0.029
AGEs (%)	85.65 ± 5.82	84.76 ± 4.79	0.55
AOPP (μmol/L)	208.34 ± 18.62	221.58 ± 12.74	0.003
FRAP (mmol/L)	982.66 ± 142.23	907.13 ± 115.87	0.04

Abbreviations: AGEs, advanced glycoprotein end product; AOPP, advanced oxidation protein product; BMI, body mass index; DBP, diastolic blood pressure; FBS, fasting blood sugar; FRAP, ferric reducing ability of plasma; HSP, heat shock protein; SBP, systolic blood pressure.

**FIGURE 1:**
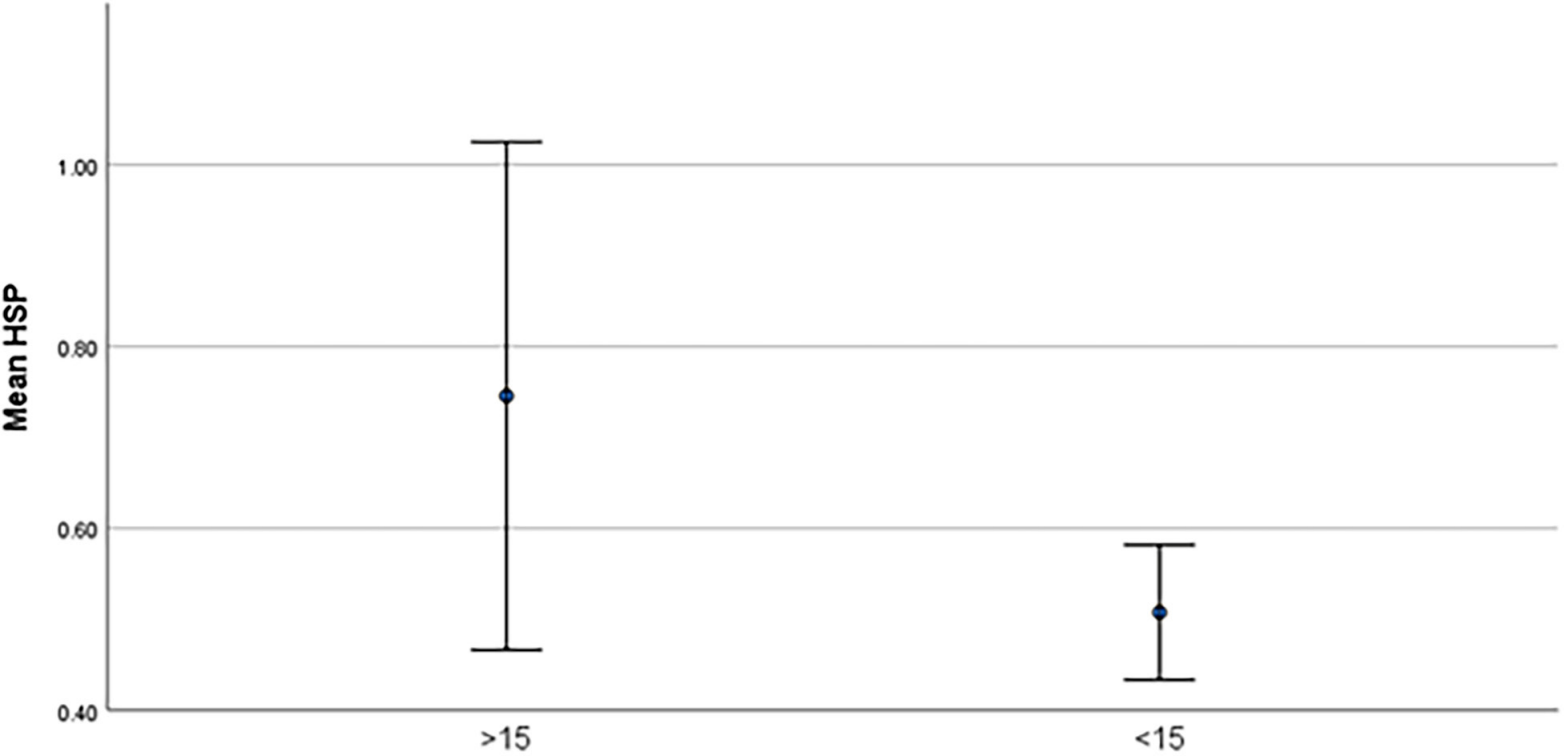
Significantly different HSP level between two groups of studied subjects.

**FIGURE 2:**
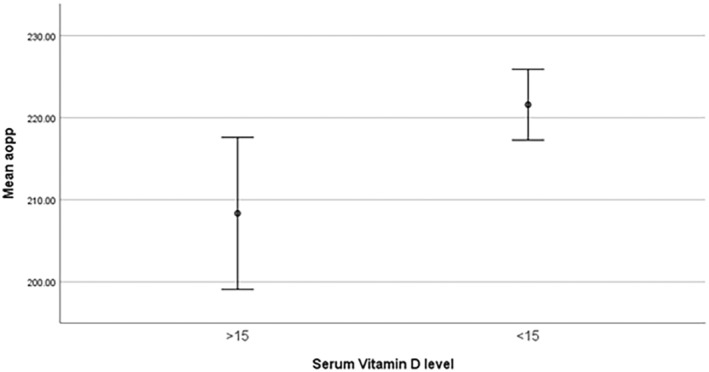
Significantly different AOPP level between two groups of studied subjects.

**FIGURE 3:**
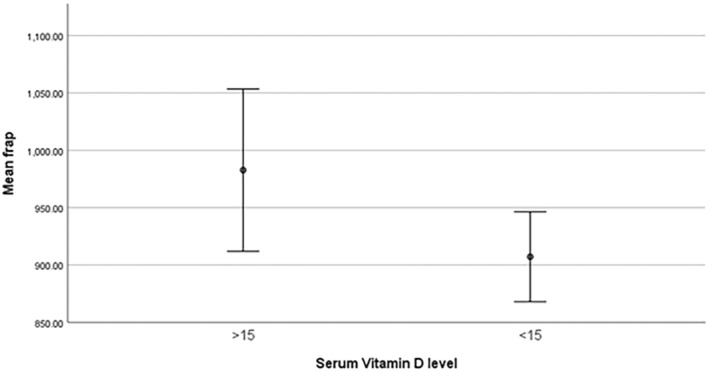
Significantly different FRAP level between two groups of studied subjects.

Figures (1, 2, 3) shows different levels of HSP, AOPP, and FRAP between two groups of studied subjects, Table 2 shows the correlation between the serum vitamin D level and OS biomarkers (AGEs, FRAP, HSP, AOPP) and other variables.

**TABLE 2:** Regression analysis of the serum vitamin D level and other variables.

Serum vitamin D level		
Variables	*β*	*p*
HSP (ng/mL)	0.391	0.003
AGEs (%)	0.077	0.579
AOPP (μmol/L)	−0.301	0.027
FRAP (mmol/L)	0.323	0.017
FBS (mg/dL)	−0.237	0.101
HbA1C (%)	−0.038	0.797
TG (mg/dL)	−0.094	0.545
Cholesterol (mg/dL)	0.089	0.560
HDL‐C (mg/dL)	−0.109	0.480
LDL‐C (mg/dL)	0.103	0.504

*Note:* After adjusting for multiple confounders such as age, sex and other metabolic markers like blood pressure and lipid profile (TG, Chol, LDL, HDL) in multivariate regression analysis, HSP remained significantly in correlation with serum vitamin D levels.

Abbreviations: AGEs, advanced glycoprotein end product; DBP, diastolic blood pressure; FBS, fasting blood sugar; FRAP, ferric reducing ability of plasma; HbA1c, Haemoglobin A1c; HDL, high‐density cholesterol; LDL‐C, low‐density lipoprotein cholesterol; SBP, systolic blood pressure.

**FIGURE 4:**
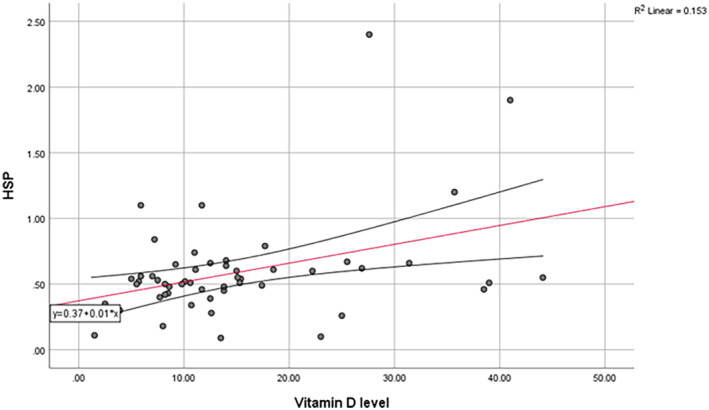
Linear correlation between serum vitamin D levels and HSP.

**FIGURE 5:**
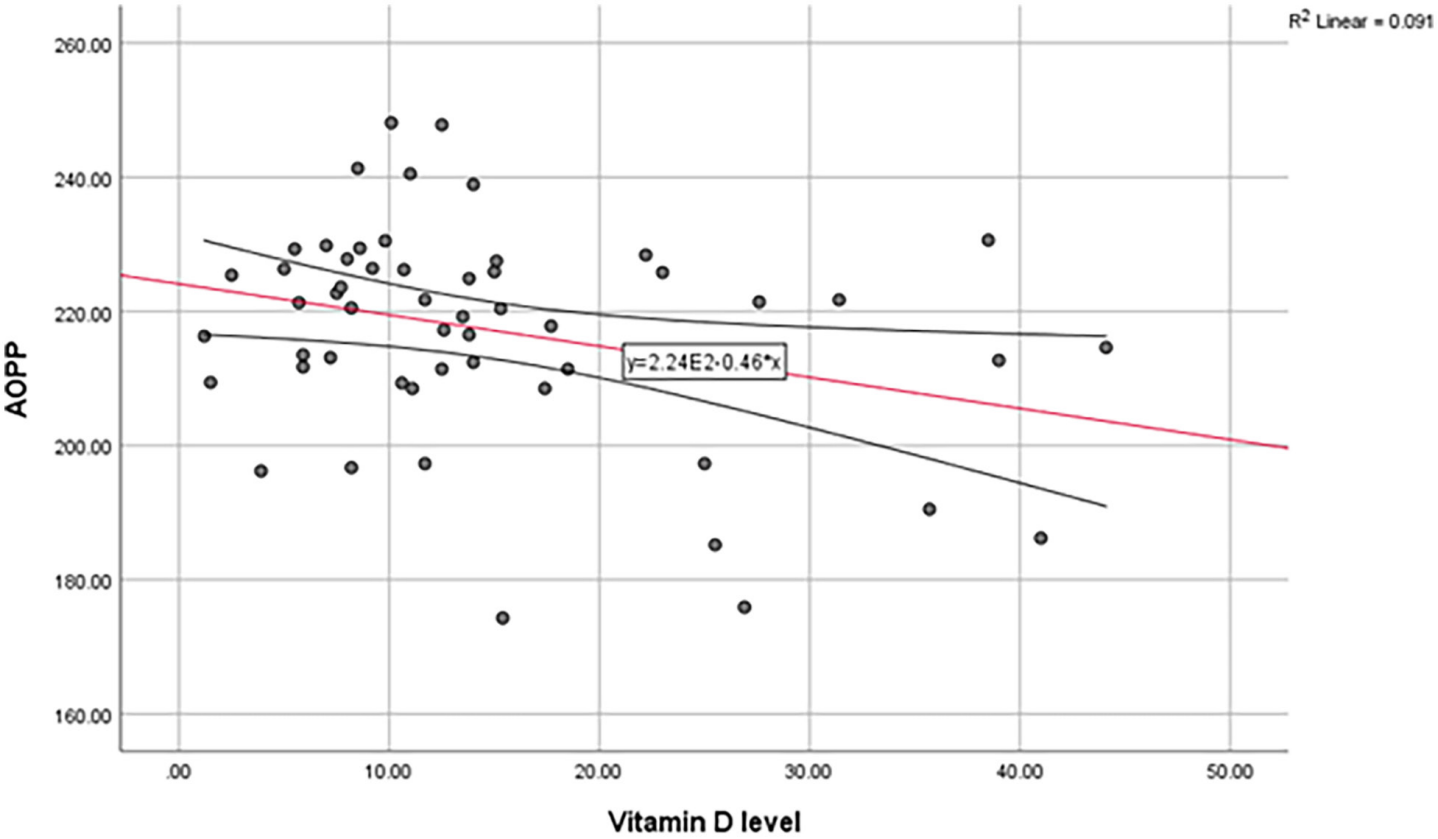
Linear correlation between serum vitamin D levels and AOPP.

**FIGURE 6:**
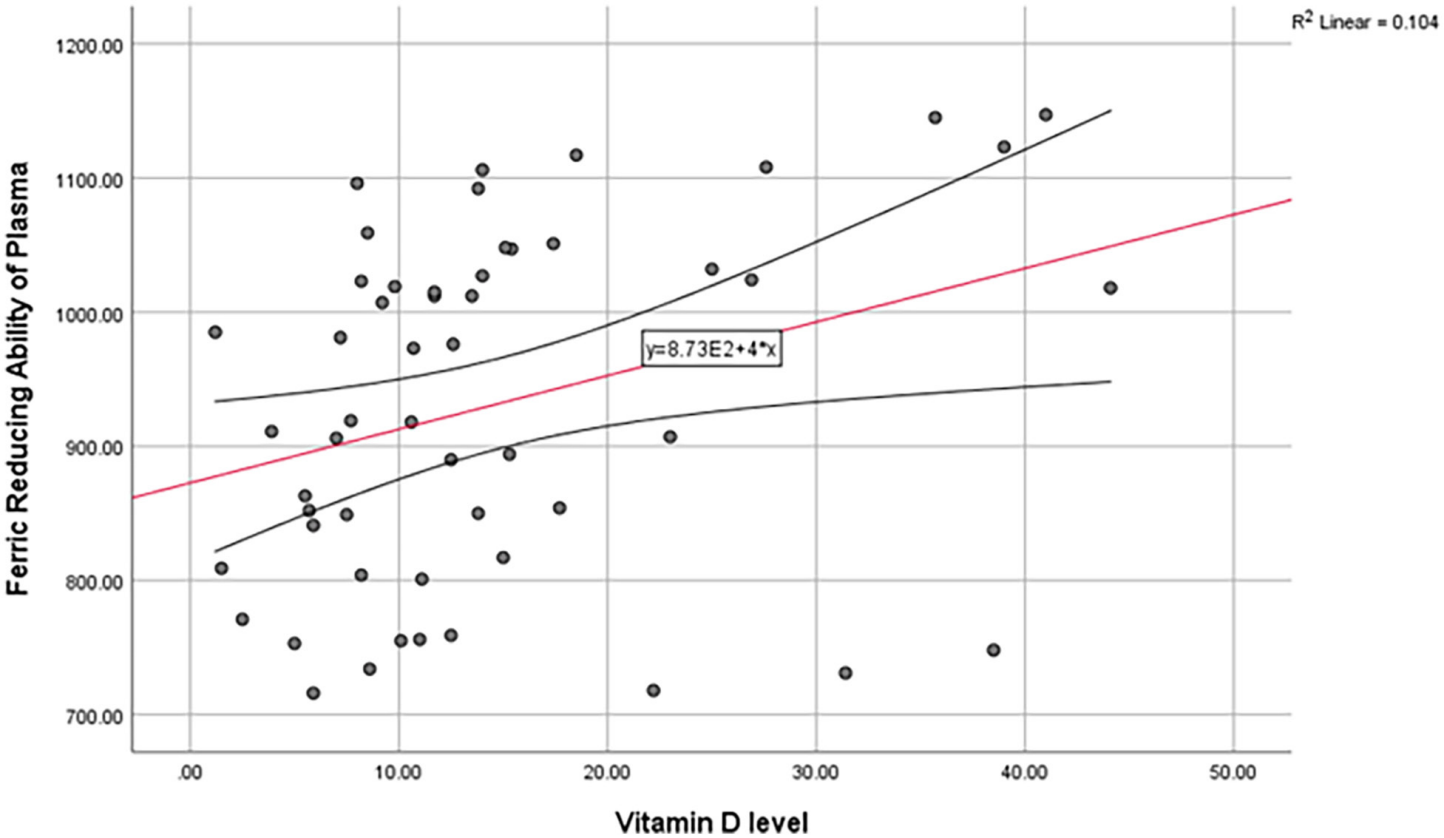
Linear correlation between serum vitamin D level and FRAP.

Vitamin D level had a positive correlation with FRAP (*r* = 0.32, *p* = 0.017) and HSP (*r* = 0.39, *p* = 0.003), but had a negative correlation with AOPP (*r* = −0.30, *p* = 0.02). The level of 2hPP also had a negative correlation with the level of vitamin D (*r* = −0.33, *p* = 0.03). (Figure 4, 5, 6) There was not any relationship between the level of vitamin D and AGEs or other variables.

Because of the significant relationship between serum vitamin D level and AOPP, FRAP and HSP in the univariate linear regression, multivariable linear regression was applied to predict these factors through serum vitamin D level:

$$\mathrm{HSP}=0.37+0.01^{*}\;\left(\text{Vitamin}\;D\;\text{level}\right)\;R^{2}=0.153$$

$$\begin{array}{c} \text{AOPP}=2.24^{*}100-0.46^{*}\left(\text{Vitamin}\;D\;\text{level}\right)\;R^{2}=0.091 \end{array}$$

$$\begin{array}{c} \text{FRAP}=8.37^{*}100+4^{*}\left(\text{Vitamin}\;D\;\text{level}\right)\;R^{2}=0.104 \end{array}$$

Finally, multiple linear regression was used to investigate the relationship between the level of vitamin D and AOPP, FRAP and HSP. After adjusting for multiple confounders such as age, sex and other metabolic markers like blood pressure and lipid profile (TG, Chol, LDL, HDL) in multivariate regression analysis, HSP remained significantly in correlation with serum vitamin D levels (*p* = 0.003).

## 4 Discussion

In this study, around 70% of the studied subjects had vitamin D (25OH vitamin D3) levels less than 15 ng/dL which is considered as vitamin D deficiency according to previous studies [31, 32]. It seems vitamin D deficiency is more prevalent among patients with T2D [1, 2, 3]. The current knowledge suggests an association between vitamin D deficiency and diabetes pathogenesis, increased insulin resistance, OS and diabetic complications [1, 2, 3, 5, 7, 8, 33]. Jung et al. [33] reported vitamin D level of less than 10 ng/mL was associated with diabetic complications such as peripheral neuropathy in male patients and diabetic nephropathy in females. According to the study of Nikooyeh et al., [8] vitamin D possesses antioxidant properties and lowers the OS level in T2D. OS provides a favourable inflammatory condition to develop diabetic complications. Some studies showed an inverse association between vitamin D and HbA1c levels and glycaemic control in patients with T2D [34, 35]. Better glycaemic control reduces diabetes progression and its complications. Vitamin D supplementation in patients with T2D is associated with significantly decreased insulin resistance and HbA1c [8, 35]. It seems vitamin D plays a significant role not only in the pathogenesis of diabetes but also in dynamically affecting diabetes control and its complications.

The present study showed a significantly decreased level of FRAP in patients with T2D and vitamin D levels less than 15 ng/dL. Also, there was a positive correlation between the levels of vitamin D and FRAP. Hisalkar et al. observed significantly decreased levels of FRAP in patients with T2D. Patients with poor glycaemic control and higher HbA1c had significantly lower levels of FRAP [36]. Antioxidant capacity was lower in vitamin D deficient patients with T2D in another study [37]. The results of several clinical trials showed vitamin D supplementation in patients with T2D and vitamin D deficiency can be a simple but important potential therapeutic intervention to improve glycaemic control and reduce the increased level of OS and its consequences [22, 38]. Decreased OS level and improved glycaemic control in patients with T2D who were treated with vitamin D supplementation can be a proof of this claim [39].

This investigation revealed that both groups of patients had increased levels of HSP70. However, what was unexpected is that patients with vitamin D deficiency had lower HSP70 levels, whereas we expected these patients to have more HSP70 levels. We hypothesised at the early stages of increased cellular OS, the iHSP70 level increases in parallel, to protect the cellular structure and function and maintenance of cellular organisation. As the OS level increases, depending on the OS intensity, a part of iHSP70 is released from the cell and increases the level of eHSP70 [40]. The level of iHSP70 and consequently the eHSP70 levels continue to rise until the cellular defence capacity is saturated. From here, despite of increasing stress intensity or chronicity, this line of cellular defence will not function efficiently and a vicious cycle begins. Therefore, although the patient will have an elevated level of HSP70, this increase is not as much as expected. As confirmation of this hypothesis, it has been observed that patients with T2D have lower levels of the HSP70 gene [41, 42, 43]. Diabetes impairs cellular antioxidant capacity against OS. These altered cellular responses to OS may contribute to increased insulin resistance in diabetes [42, 43]. In animal studies, restoring HSP70 deficiencies in diabetic conditions can improve glucose tolerance significantly [41]. Former studies have suggested an association between the increased intracellular HSP70 expression and increased intracellular VDR expression [12, 13]. Regarding to antioxidant activity that both HSP70 and vitamin D have shown, and the positive correlation between vitamin D and HSP70 levels, it seems these two antioxidant systems share at least a part of their functional pathway.

It is pertinent to note that in this study, a decreased level of AOPP was seen in those patients with vitamin D levels more than 15 ng/dL. There was a negative correlation between vitamin D and AOPP levels. Similar results had been reported in previous studies [44, 45]. Vitamin D supplementation was accompanied by better glycaemic control and decreased AOPP level as an OS marker [45]. As diabetes progresses and vascular complications such as nephropathy develop, the AOPP level increases and patients with diabetic vascular complications have significantly higher levels of AOPP compared with patients with non‐complicated diabetes [44, 46].

This research did not show any significant difference between the level of AGEs in the two groups of diabetes compared with each other. However, due to the high costs of the laboratory kits, our study lacked a healthy control group and it would be better to compare both patient groups with a healthy control group. No correlation was seen between vitamin D and AGE levels. Gradinaru et al. [44] showed increased levels of AGEs in patients with T2D. In normal metabolic milieu, the reaction of reducing sugars with lipids, proteins and DNA molecules produces AGEs as by‐products, but under hyperglycaemia and OS, these reactions and AGE production are accentuated [47]. Although the antioxidant effect of vitamin D, Šebeková et al. [48] reported no correlation between vitamin D and AGEs levels. Currently, according to available studies, it seems that despite the involvement of both vitamin D and AGEs in antioxidant–oxidant processes, but they act in different pathways.

Because of a shortage of budget, we had to limit our sample size. A larger sample size could provide more precise results. Also, it would be better to study two groups of healthy people with and without vitamin D deficiency as controls. Because of a lack of control groups, we had to compare the amount of the measured biomarkers with their normal level based on previous studies. Single centre nature was another limitation of the study.

## 5 Conclusion

Vitamin D deficiency is common in patients with diabetes; this study highlights the relationship between vitamin D level and OS biomarkers in patients with T2D. A significantly decreased level of FRAP in patients with T2D and vitamin D levels less than 15 ng/dL, and also, a positive correlation between the levels of vitamin D and FRAP were observed in this research.

## Author Contributions

**Nazanin Hashemi:** data curation (equal), formal analysis (equal), methodology (equal), writing–original draft (equal). **Sahar Karimpour Reyhan:** methodology (equal), validation (equal), writing–original draft (equal), writing–review and editing (equal). **Reihane Qahremani:** formal analysis (equal), methodology (equal), writing–original draft (lead), writing–review and editing (equal). **Kiana Seifouri:** validation (equal), writing–original draft (equal), writing–review and editing (equal). **Meraj Tavakoli:** writing–original draft (equal), writing–review and editing (equal). **Seyed Arsalan Seyedi:** formal analysis (equal), visualization (equal). **Farahnaz Ghaemi:** data curation (equal), investigation (equal). **Mahsa Abbaszadeh:** data curation (equal), methodology (equal), visualization (equal). **Manouchehr Nakhjavani:** conceptualization (equal), project administration (equal), supervision (equal), writing–review and editing (equal). **Alireza Esteghamati:** conceptualization (equal), project administration (equal), supervision (equal). **Hossein Mirmiranpour:** investigation (equal), visualization (equal). **Soghra Rabizadeh:** formal analysis (lead), project administration (equal), supervision (lead), validation (equal), writing–review and editing (equal). All authors contributed to the manuscript.

## Conflicts of Interest

The authors declare no conflicts of interest.

## Data Availability

The data are available with the authors and will be provided upon request.
